# Facile solution-phase synthesis of γ-Mn_3_O_4 _hierarchical structures

**DOI:** 10.1186/1752-153X-1-8

**Published:** 2007-03-17

**Authors:** Zhengcui Wu, Kuai Yu, Yaobin Huang, Cheng Pan, Yi Xie

**Affiliations:** 1 Department of Nanomaterials and Nanochemistry, Hefei National Laboratory for Physical Sciences at Microscale, University of Science and Technology of China, Hefei 230026, P. R. China; 2 Anhui Key Laboratory of Functional Molecular Solids, College of Chemistry and Materials Science, Anhui Normal University, Wuhu 241000, P. R. China

## Abstract

**Background:**

A lot of effort has been focused on the integration of nanorods/nanowire as building blocks into three-dimensional (3D) complex superstructures. But, the development of simple and effective methods for creating novel assemblies of self-supported patterns of hierarchical architectures to designed materials using a suitable chemical method is important to technology and remains an attractive, but elusive goal.

**Results:**

The hierarchical structure of Mn_3_O_4 _with radiated spherulitic nanorods was prepared via a simple solution-based coordinated route in the presence of macrocycle polyamine, hexamethyl-1,4,8,11-tetraazacyclotetradeca-4,11-diene (CT) with the assistance of thiourea as an additive.

**Conclusion:**

This approach opens a new and facile route for the morphogenesis of Mn_3_O_4 _material and it might be extended as a novel synthetic method for the synthesis of other inorganic semiconducting nanomaterials such as metal chalcogenide semiconductors with novel morphology and complex form, since it has been shown that thiourea can be used as an effective additive and the number of such water-soluble macrocyclic polyamines also makes it possible to provide various kinds of ligands for different metals in homogeneous water system.

## Background

Recently, a lot of effort has been focused on the integration of nanorods/nanowire as building blocks into three-dimensional (3D) complex superstructures. There are a variety of methods for different materials to construct 3D superstructures, among which hierarchical α-MnO_2_, ZnO, CaCO_3 _nanostructures, penniform BaWO_4 _nanostructures and dandelion-like CuO nanostructures have been successfully prepared [1-5]. These results not only provide feasible ways to assemble 1D nanostructures for future microscale functional devices but also offer opportunities to explore their novel collective properties.

Considerable research has focused on trimanganese tetroxide due to its catalytic and soft magnetic properties in recent years. It has been used as a catalyst for several processes, e.g., the oxidation of methane and carbon monoxide [6,7], the decomposition of nitrogenoxides [8], the selective reduction of nitrobenzene [9], and the catalytic combustion of organic compounds at temperatures of 373–773 K [10]. Mn_3_O_4 _is often synthesized by the high-temperature calcinations of manganese powders or manganese oxides with a higher valence of manganese, hydroxides, and hydroxyoxides, or oxysalts of manganese [11-14]. Using carbonaceous polysaccharide microspheres as templates, Mn_3_O_4 _hollow spheres were also prepared [15]. Other solution-based methods based on the oxidation of the Mn(II) compound or the reduction of KMnO_4 _have also been employed to prepare Mn_3_O_4 _nanoparticles or nanorods [16-26]. Colloidal Mn_3_O_4 _monodisperse nanoparticles or nanorods were also prepared from thermal decomposition of precursor [Mn(acac)]_2 _(acac = acetylacetonate) in oleylamine, thermolysis of Mn(HCOO)_2 _in oleylamine, or thermally induced crystal growth processes from MnCl_2 _in oleic acid and oleylamine under argon atmosphere [27-29]. The development of simple and effective methods for creating novel assemblies of self-supported patterns of hierarchical architectures to designed materials using a suitable chemical method is important to technology and remains an attractive, but elusive goal.

Macrocyclic polyamine, a kind of cyclic ligand that can completely enclose metal ions, is usually of interest to chemists from a synthetic viewpoint or for its structure and coordination with metallic ions. Considering the strong coordination ability of macrocyclic polyamines with many metallic ions, the number of such compounds and their convenience for large-scale production, which may be applied to control the release of metallic ions at elevated temperature that are propitious to building complex architectures of inorganic crystals, we successfully designed and synthesized β-Ni(OH)_2 _flower-like pattern using hexamethyl-1,4,8,11-tetra-azacyclotetradeca-4,11-diene (CT), in a water system [30]. This was the first report on the construction of inorganic nanomaterials with macrocycle polyamines. This success led us to use a macrocyclic polyamine to direct the growth of other inorganic crystals with novel morphologies and architectures. However, a recent study convinced us that thiourea could be used as an additive to control the morphology of the inorganic material [31]. The addition of thiourea in the hydrolysis of K_2_SnO_3_·3H_2_O in an ethanol-H_2_O mixed solvent system resulted in nearly 100% hollow SnO_2 _spheres with increased product yield and morphological yield compared with that of the absence of thiourea, which is very different from the commonly accepted view that thiourea is a mild sulfur source. This interesting effect of thiourea inspired us to synthesize other metal oxides with complex morphologies using thiourea as an additive. It should be pointed out that the concentration ratio of the additive thiourea and of K_2_SnO_3_·3H_2_O was about 7:1 in the above-mentioned literature (with 0.1 M thiourea and 15 mM K_2_SnO_3_·3H_2_O, respectively). To generalize the morphological control of thiourea on complex morphogenesis of metal oxides, herein, we added thiourea in the synthesis of Mn_3_O_4 _hierarchical structure via a simple solution-based coordinated route in the presence of CT, and found that low dose of thiourea also has an important effect on the morphological control. A new example for the morphogenesis of hierarchical structure of Mn_3_O_4 _with radiated spherulitic nanorods in the presence of CT with the assistance of thiourea as an additive will be demonstrated in this paper. The products display an elegant morphology resembling a thorny sphere, which is rarely reported for Mn_3_O_4_, providing us another opportunity for exploring the properties dependent on their morphologies.

## Results and Discussion

**Figure 1:**
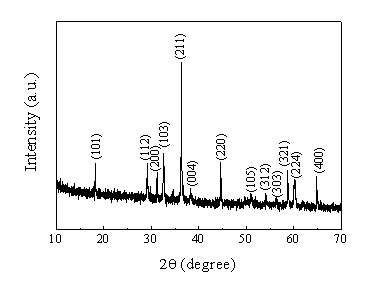
XRD pattern of the as-prepared Mn_3_O_4 _spheres with radiated spherulitic nanorods. The diffraction peaks can be indexed to tetragonal phase of γ-Mn_3_O_4_, indicating the high crystallinity.

The crystal structure and phase composition of Mn_3_O_4 _products were first characterized using X-ray powder diffraction (XRD). Figure 1 displays a representative XRD pattern of the as-prepared Mn_3_O_4 _samples, suggesting their high crystallinity. The diffraction peaks can be readily indexed to the tetragonal phase of γ-Mn_3_O_4 _with lattice parameters of a = 5.749 Å and c = 9.432 Å which are consistent with the standard values (JCPDS Card, No. 80-0382).

**Figure 2:**
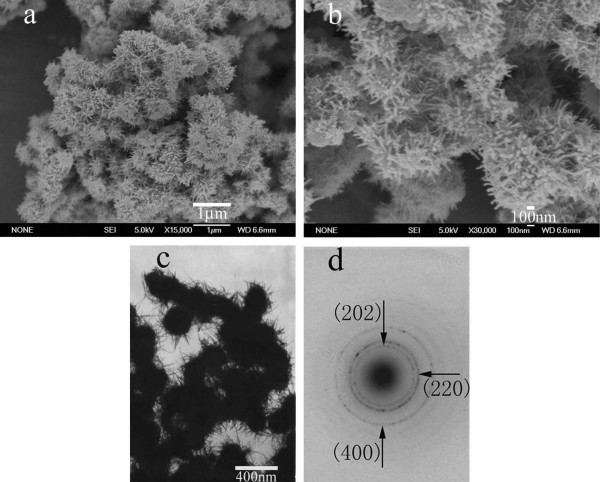
Typical FESEM images and TEM images of the γ-Mn_3_O_4 _spheres with radiated spherulitic nanorods. **a**: FESEM image at a low magnification, indicating that the γ-Mn_3_O_4 _spheres can be fabricated on a large scale. **b**: FESEM image at a high magnification, revealing the nanorods were fixed on the surfaces of the spheres. **c**: TEM image of the γ-Mn_3_O_4 _radiated spherulitic nanorods. **d**: The corresponding SAED pattern of the γ-Mn_3_O_4 _products.

The submicrometer-sized hierarchical structures of Mn_3_O_4 _were successfully synthesized on a large scale, as revealed in Figure 2a where a panoramic Field Emission Scanning Electron Microscope (FESEM) image of the product is displayed, revealing that the sample consisted of radiated spherulitic nanorods with yield of nearly 100% in the diameter about 400 nm. The magnified FESEM image (Figure 2b) showed that nanorods with uniform diameters around 30 nm were fixed on the surfaces of the spheres, and they were densely packed and radiated spherulitic. The morphology and structure of the products are further detected by Transmission Electron Microscopy (TEM) and Selected Area Electron Diffraction (SAED). Figure 2c shows the representative TEM image of the Mn_3_O_4 _hierarchical structures, which further confirmed the products were radiated spherulitic nanorods. Figure 2d shows the corresponding SAED pattern, which also demonstrates the tetragonal structure of Mn_3_O_4_.

**Figure 3:**
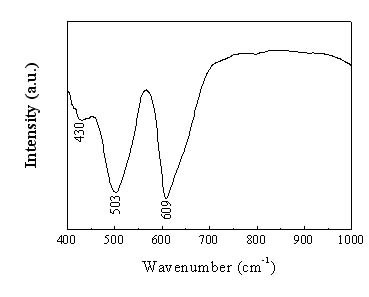
FTIR spectrum of as-prepared Mn_3_O_4 _radiated spherulitic nanorods.

Figure 3 shows Fourier Transform Infrared (FTIR) Spectrum of the as-prepared γ-Mn_3_O_4 _products, displaying a notable resemblance to those of Mn_3_O_4 _obtained in previous studies [32,12]. In the region from 650 to 500 cm^-1 ^of the observed spectrum, two absorption peaks were observed at 609 and 503 cm^-1^, which may be associated with the coupling modes between the Mn-O stretching modes of tetrahedral and octahedral sites. In the region from 500 to 400 cm^-1^, the absorption peak at 430 cm^-1 ^was assigned as the band-stretching mode of the octahedral sites; the displacement of the Mn^2+ ^ions in tetrahedral sites was negligible. Therefore, the FTIR spectra further confirm the formation of Mn_3_O_4 _products.

**Figure 4:**
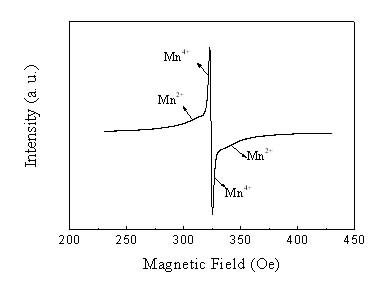
ESR spectrum of the Mn_3_O_4 _radiated spherulitic nanorods.

The as-prepared product of γ-Mn_3_O_4 _was further examined using Electron Spin Resonance (ESR). Since ESR can be used to detect paramagnetically isolated species and give information about the coordination of isolated sites, it can be used to detect Mn^2+ ^and Mn^4+^. Theoretically, Mn_3_O_4 _contains Mn^4+ ^(calculated as 39–40% MnO_2_) with the rest of the Mn as Mn^2+^. Therefore, the ESR should contain the signals for both Mn^2+ ^and Mn^4+^. Figure 4 shows the ESR spectrum of the sample, demonstrating a similar result with the literature [12]. A typical signal of Mn^2+ ^with a scarcely resolved hyperfine structure accompanied by poignant signal of Mn^4+ ^can be seen, which corroborates the valences of Mn in the sample.

**Figure 5:**
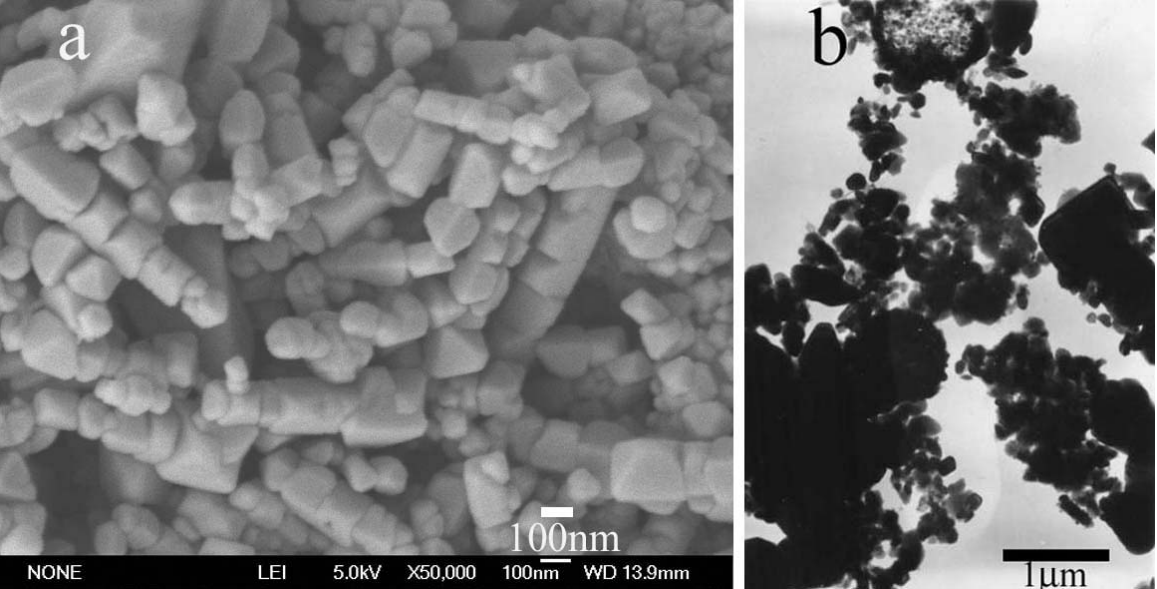
TEM or FESEM images of γ-Mn_3_O_4 _products obtained under different reaction conditions. **a**. FESEM image of 0.084 g MnSO_4_·H_2_O and 5.0 g CT at 210°C without thiourea. **b**. TEM image of 0.084 g MnSO_4_·H_2_O and 5.0 g CT at 210°C with 0.039 g urea.

To understand the formation mechanism of our Mn_3_O_4 _products, the experiment was repeated without thiourea in the reaction system and with other parameters being the same. The product formed was polyhedron Mn_3_O_4 _nanoparticles as shown in Figure 5a (see Figure S1a for the corresponding XRD pattern in additional file 1), therefore, the formation of Mn_3_O_4 _radiated spherulitic nanorods with yield of nearly 100% is obviously a result of the addition of thiourea. To further make clear the role of thiourea, urea was used instead in the synthetic mixtures with other parameters remaining the same. Only sparse hollow spheres accompanied by abundant irregular particles were obtained as shown in Figure 5b (see Figure S1b for the XRD pattern in additional file 1), indicating that the addition of urea did not result in the formation of uniform γ-Mn_3_O_4 _nanostructures. This is different from the above-mentioned literature in that the added urea also improved the product yield and morphological yield of hollow SnO_2 _spheres [31]. This confirmed that low doses of thiourea could play a decisive and exclusive role in the formation of Mn_3_O_4 _hierarchical structure.

The possible chemical reaction in the solution can be described as follows. At the beginning of reaction, Mn^2+ ^cations were released slowly from the complex of Mn^2+^-CT at elevated temperature, that is to say, on this alkalescent and heating condition, the Mn(CT)^2+ ^is unstable and the following reactions occur:



Mn(CT)^2+ ^→ Mn^2+^+CT

6Mn^2+^+O_2_+12OH^- ^→ 2Mn_3_O_4_+6H_2_O

It can be reasonably assumed that the release of S^2- ^ions from the decomposition of thiourea in the initial reaction stage remarkably affects the nucleation and subsequently growth processes on γ-Mn_3_O_4 _products. This is a competitive reaction between the formation of MnS and Mn_3_O_4 _at elevated temperature in our reaction system and the formation of Mn_3_O_4 _had larger tendency than that of MnS in the presence of abundant alkalescent macrocyclic polyamine. However, the presence of S^2- ^anions decreased the formation and growth speed of Mn_3_O_4 _seeds, which was in favor of the formation of uniform hierarchical products. By contrast, the addition of urea quickened the hydrolyzation of Mn^2+ ^that sped up the nucleation and growth process of Mn_3_O_4_, resulting in irregular particles. The slow formation and growth of Mn_3_O_4 _in competition with S^2- ^ions at the expense of destroying the coordination between Mn^2+ ^and CT was favorable for the formation of hierarchical structure. Here, the coordination of CT with Mn^2+ ^and subsequent absorption of CT on Mn_3_O_4 _seeds cannot be overlooked on the morphology formation of the product. Being a cyclic ligand that can completely enclose metal ions, it is well known that tetradentate macrocyclic ligands coordinate in a square planar fashion, which significantly affects the growth of Mn_3_O_4 _hierarchical structure. As mentioned above, Mn_3_O_4 _seeds gradually formed in competition with S^2- ^ions at the expense of destroying the coordination between Mn^2+ ^and CT, in the subsequent growth step, since the release of S^2- ^ions reduced the growth speed of the product, the role of CT gradually became dominant. The coordination of tetradentate macrocyclic ligands in a square planar fashion and subsequent selective absorption on Mn_3_O_4 _seeds led to the growth of the particles in an oriented direction, thus forming the final hierarchical structure.

**Figure 6:**
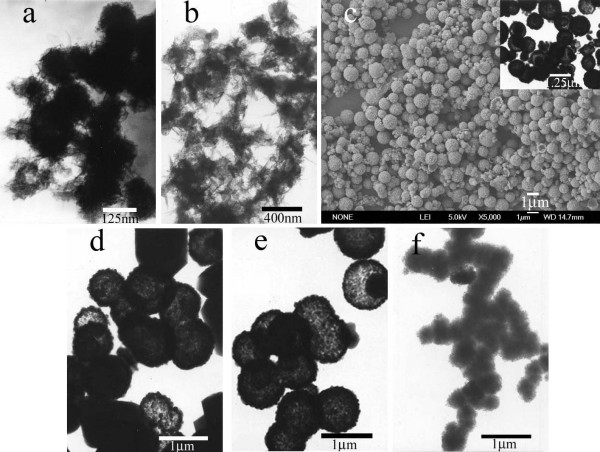
TEM or FESEM images of γ-Mn_3_O_4 _products obtained under different reaction conditions. **a**. TEM image of 0.084 g MnSO_4_·H_2_O, 5.0 g CT and 0.040 g thiourea at 180°C. **b**. TEM image of 0.084 g MnSO_4_·H_2_O, 5.0 g CT and 0.040 g thiourea at 240°C. **c**. FESEM image of 5.0 g CT, 0.126 g MnSO_4_·H_2_O and correspondingly varied thiourea at 210°C. The inset in Figure 6c was the corresponding TEM image. **d**. TEM image of 5.0 g CT, 0.21 g MnSO_4_·H_2_O and correspondingly varied thiourea at 210°C. **e**. TEM image of 0.084 g MnSO_4_·H_2_O, 0.040 g thiourea and 4.0 g CT at 210°C. **f**. TEM image of 0.084 g MnSO_4_·H_2_O, 0.040 g thiourea and 6.7 g CT at 210°C.

The reaction temperature and the concentration of the reactants on the morphology of the γ-Mn_3_O_4 _products were also investigated. Kinetics theory implies that temperature greatly influences the rate of hydrolysis, nucleation as well as on the growth processes. The temperature effect is apparent by comparing Figure 6a and Figure 6b. The product synthesized at 180°C mostly consists of spheres with shorter nanorods on their surfaces (Figure 6a), while those prepared at 240°C showed many distorted and mutually joined spheres (Figure 6b), which may be partly due to the magnetism of the products at high temperature. Similar spontaneous aggregation and assembly phenomena were also observed on other magnetic materials [33,34]. When the amount of MnSO_4_·H_2_O was increased to 0.126 g and that of thiourea increased correspondingly while maintaining a constant reaction temperature of 210°C, the morphologies of Mn_3_O_4 _showed a mixture of core-shell and hollow spheres as shown in Figure 6c (see Figure S1c for the XRD patterns in additional file 1). When the amount of MnSO_4_·H_2_O was further increased to 0.21 g with a correspondingly increase in that of thiourea at 210°C, the product had several morphologies including core-shell, hollow spheres and a small quantity of polyhedron, shown in Figure 6d (see Figure S1d for the XRD patterns in additional file 1). The formation of core-shell and hollow spheres is rarely reported relating to Mn_3_O_4 _and may be due to the typical inside-out Ostwald ripening effect [31,35]. The existence of polyhedron in Figure 6d may be attributed to the different nucleation processes in higher concentration of reactants. When the mass of CT varied from 4.0 g to 6.7 g while keeping MnSO_4_·H_2_O and thiourea constant at 0.084 g and 0.040 g, the morphology of γ-Mn_3_O_4 _varied from the mixture of core-shell and hollow spheres to small flocky solid spheres as shown in Figure 6e and Figure 6f, respectively. The result of decreasing the concentration of CT (Figure 6e) was similar to that of increasing concentration of MnSO_4_·H_2_O and thiourea (shown in Figure 6c), which confirmed that the concentration of the reactants was an important factor to the morphology formation of the final products. As the concentration of CT increased enough, the absorption of CT on the seeds of the Mn_3_O_4 _products was more complete, which would restrain the growth of the nanorods, leading to small flocky spheres. The formation of γ-Mn_3_O_4 _architectures relates to the experimental parameters as well as thermodynamics and kinetics control on the reaction and interaction between metal salts, CT and thiourea, which are well known factors influencing crystal growth. The kinetics is modulated by adjusting the temperature and the concentration of reagents, which control the hydrolysis rate and ratio, thus controlling the nucleation and growth processes. The above experimental results suggest that it is possible to control and tune the shape of γ-Mn_3_O_4 _nanostructures by controlling the kinetic parameters of the reaction process, that is, the reaction temperature and the concentration of reactants.

It is easily supposed that macrocycle polyamine metal complexes control the release speed of Mn^2+ ^ions and subsequently affect the nucleation and growth process of the product together with thiourea. At elevated temperature, S^2- ^anions were also released from the decomposition of CS(NH_2_)_2_, however, MnS was not formed in the existence of abundant alkalescent macrocycle polyamine. As an additive, thiourea played an important role on the shape control of γ-Mn_3_O_4_, which still needed more detailed and systematic work to provide evidence to make clear the precise functions of thoiurea in the hierarchical Mn_3_O_4 _materials. However, the synergistic effect of macrocycle polyamine and thiourea on the shape control of γ-Mn_3_O_4 _nanostructures was proved.

## Conclusion

In conclusion, γ-Mn_3_O_4 _hierarchical nanostructures composed of radiated spherulitic nanorods, core-shell and hollow spheres have been successfully prepared in high yield using a macrocycle polyamine as metal ion ligand and alkalescent source with the assistance of thiourea as an additive in a water system. This approach opens a new and facile route for the morphogenesis of Mn_3_O_4 _material and it might be extended as a novel synthetic method for the synthesis of other inorganic semiconducting nanomaterials such as metal chalcogenide semiconductors with novel morphology and complex form, since it has been shown that thiourea can be used as an effective additive and the number of such water-soluble macrocyclic polyamines also makes it possible to provide various kinds of ligands for different metals in homogeneous water system.

## Experimental

All chemicals were analytic grade purity and used as received without further purification. The macrocyclic polyamine, hexamethyl-1,4,8,11-tetra- azacyclotetradeca-4,11-diene (CT) was synthesized using methanol, ethylene diamine anhydrous, hydrobromic acid and acetone as reactants according to the literature [36]. Then, in a typical synthesis, 0.084 g MnSO_4_·H_2_O was put in a beaker, and 40 mL distilled water was added, then 5.0 g CT was added into the beaker under stirring. Finally, 0.040 g CS(NH_2_)_2 _was added. The mixture was stirred for a further 15 minutes, then the transparent solution was put in a 50 mL Teflon-sealed autoclave and maintained at 210°C for 6 h. The brown fluffy product floating on the solution was collected by centrifugation of the mixture, washed three times with distilled water and ethanol, and finally dried in a vacuum at 50°C for 10 h.

The structure of the samples obtained was characterized with the XRD pattern, which was recorded on a Rigaku Dmax diffraction system using a Cu Kα source (λ = 1.54187 Å). The scanning electron microscopy (SEM) images were taken with a JEOL-JSM-6700F field emission scanning electron microscope (FESEM, 15 kV). Transmission electron microscopy (TEM) images and the corresponding selected area electron diffraction (SAED) patterns were obtained with Hitachi 800 system at 200 kV. The Fourier transform infrared (FTIR) spectroscopic study was carried out with a MAGNA-IR 750 (Nicolet Instrument Co.) at room temperature with the sample in a KBr medium. The electron spin resonance (ESR) spectrum was recorded using a Bruker model ER-200D-SRC with the microwave frequency of 9.067 GHz at room temperature.

## Authors' contributions

This work was prepared in the research group of Professor Dr. YX. ZCW participated in the design and presiding the experiments and drafted the manuscript. KY carried out the TEM characterization and participated in the discussion of the manuscript. YBH and CP participated in the experiments. This project was based on the idea and realized under the guidance and consultation of Professor Dr. YX.

## Supplementary Material

Additional file 1 Additional file 1 for the XRD patterns of the Mn_3_O_4 _products synthesized at different reaction condition. Click here for file
