# Supplementary material for: Facile solution-phase synthesis of γ-Mn3O4 hierarchical structures
Source: Chem Cent J. 2007 Mar 17;1:8. doi: 10.1186/1752-153X-1-8 (PMC1994067; doi:10.1186/1752-153X-1-8)

# Additional file 1

**Facile solution-phase synthesis of γ-Mn_3_O_4_ hierarchical structures**

# Zhengcui Wu^1, 2^, Kuai Yu^1^, Yaobin Huang^1^, Cheng Pan^1^, Yi Xie^1, *^

1 Department of Nanomaterials and Nanochemistry, Hefei National Laboratory for Physical Sciences at Microscale, University of Science and Technology of China, Hefei 230026, P. R. China

2 Anhui Key Laboratory of Functional Molecular Solids, College of Chemistry and Materials Science, Anhui Normal University, Wuhu 241000, P. R. China

Figure S 1: XRD patterns of the Mn_3_O_4_ products synthesized at different reaction condition, which still can be indexed to tetragonal phase of γ-Mn_3_O_4_.

(**a**, **b**) XRD patterns of keeping 0.084g MnSO_4_·H_2_O and 5.0g CT at 210℃ without the addition of thiourea and with the addition of 0.039g urea, respectively. (**c**, **d**) XRD patterns of keeping 5.0g CT at 210℃ with 0.126g and 0.21g MnSO_4_·H_2_O and correspondingly varied thiourea, respectively.


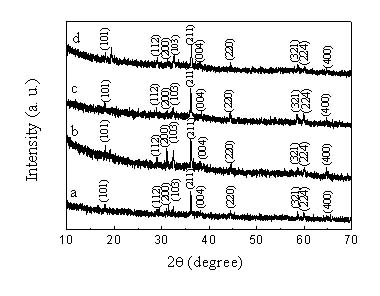

Supplement: Additional file 1 — for the XRD patterns of the Mn3O4 products synthesized at different reaction condition. [file 1752-153X-1-8-S1.doc]
